# Retinoic acid delays initial photoreceptor differentiation and results in a highly structured mature retinal organoid

**DOI:** 10.1186/s13287-022-03146-x

**Published:** 2022-09-16

**Authors:** Carla Sanjurjo-Soriano, Nejla Erkilic, Krishna Damodar, Hassan Boukhaddaoui, Michalitsa Diakatou, Marcela Garita-Hernandez, Daria Mamaeva, Gregor Dubois, Zhour Jazouli, Carla Jimenez-Medina, Olivier Goureau, Isabelle Meunier, Vasiliki Kalatzis

**Affiliations:** 1 Institute for Neurosciences of Montpellier (INM), Univ Montpellier, Inserm, Montpellier, France; 2 National Reference Centre for Inherited Sensory Diseases, Univ Montpellier, CHU, Montpellier, France; 3 Institut de La Vision, Sorbonne Université, Inserm, CNRS, Paris, France; 4 Present Address: Ocular Genomics Institute, Department of Ophthalmology, Massachusetts Eye and Ear, Harvard Medical School, Boston, MA USA

**Keywords:** Induced pluripotent stem cells, Retinal organoids, Retinoic acid, Photoreceptors, Rods, Cones

## Abstract

**Background:**

Human-induced pluripotent stem cell-derived retinal organoids are a valuable tool for disease modelling and therapeutic development. Many efforts have been made over the last decade to optimise protocols for the generation of organoids that correctly mimic the human retina. Most protocols use common media supplements; however, protocol-dependent variability impacts data interpretation. To date, the lack of a systematic comparison of a given protocol with or without supplements makes it difficult to determine how they influence the differentiation process and morphology of the retinal organoids.

**Methods:**

A 2D-3D differentiation method was used to generate retinal organoids, which were cultured with or without the most commonly used media supplements, notably retinoic acid. Gene expression was assayed using qPCR analysis, protein expression using immunofluorescence studies, ultrastructure using electron microscopy and 3D morphology using confocal and biphoton microscopy of whole organoids.

**Results:**

Retinoic acid delayed the initial stages of differentiation by modulating photoreceptor gene expression. At later stages, the presence of retinoic acid led to the generation of mature retinal organoids with a well-structured stratified photoreceptor layer containing a predominant rod population. By contrast, the absence of retinoic acid led to cone-rich organoids with a less organised and non-stratified photoreceptor layer.

**Conclusions:**

This study proves the importance of supplemented media for culturing retinal organoids. More importantly, we demonstrate for the first time that the role of retinoic acid goes beyond inducing a rod cell fate to enhancing the organisation of the photoreceptor layer of the mature organoid.

**Supplementary Information:**

The online version contains supplementary material available at 10.1186/s13287-022-03146-x.

## Background

There are 450 million people worldwide estimated to be visually impaired [1]. Major causes are inherited retinal diseases (IRDs), age-related macular degeneration or glaucoma, which are due to the degeneration of the light-sensing photoreceptors, the supporting retinal pigment epithelium (RPE) or the retinal ganglion cells (RGCs), respectively [2, 3]. These diseases are clinically and genetically heterogeneous, making the development of efficient therapies highly challenging [3]. Encouragingly, the first retinal gene therapy drug, specific to IRDs caused by *RPE65* mutations, is on the market [4]. However, with over 270 causative IRD genes [5], there is still an unmet need for novel therapies that would benefit more patients.

An obstacle for the development of efficient treatments has been a lack of appropriate models. Whilst animal models have greatly contributed to the IRD field, they do have limitations because they do not always mimic key aspects of human retinal pathophysiology. This is most likely due to genetic and anatomical differences; therefore, there was a need for the development of human IRD models. Today, thanks to the recent technological revolutions of human induced pluripotent stem cell (hiPSC) generation [6] and organoid differentiation [7], disease modelling and regenerative medicine for the retina have reached the forefront of translational research [8–18].

The first protocols for the differentiation of hiPSCs to a retinal lineage and to photoreceptor precursor cells comprised two-dimensional (2D) adherent culture systems [19–21]. Subsequently, the development of three-dimensional (3D) culture systems resulted in the production of self-organising optic vesicle-like structures that recreate the architecture of the neural retina (NR) [8–11]. These organoid models recapitulate the main steps of retinal development and, at maturity, contain a layer of photoreceptors with outer segment (OS)-like structures. Over the last decade, these protocols have been used to produce retinal organoids [13, 22–28] for disease modelling [12, 15, 17, 29, 30] and as a source of transplantable cells [14, 16, 18, 31]. However, there are still several challenges. A first challenge is the highly variable differentiation potential of hiPSC, or even hPSC, lines and morphology of derived retinal organoids within a given line [13, 32–34]. This variability can be misleading and seriously impact data interpretation. A second challenge is the low efficiency of organoid production [33]. Consequently, multiple cultures are required to generate a sufficient number of retinal organoids per study, which is labour-intensive and financially draining. Therefore, there is a need to consolidate robust protocols that will produce retinal organoids with maximum efficiency and minimum heterogeneity.

This gives rise to a third challenge though, as each modification introduced to optimise retinal orgnanoid differentiation results in protocol-dependent variability. The addition of supplements, small molecules or signalling factors to improve retinal differentiation in long-term culture has become a common and extensive practice [8, 9, 12, 14, 15, 17]. However, to date, a systematic comparison of a given protocol, with or without supplements and using the same cell line, has never been performed making it difficult to draw any firm conclusions. We thus differentiated the same hiPSC line into organoids using the same protocol with or without the most commonly used supplements, most notably retinoic acid (RA), and studied the effects up to 225 days of culture. In this way, we determined the direct impact on the differentiation process and morphology of the mature retinal organoids.

## Methods

### hiPSC culture

We used a hiPSC line that we previously generated [35] and that readily differentiates into retinal organoids [36]. For differentiation Protocol 1 (see below), hiPSCs were maintained on 35 mm cell culture dishes coated with truncated recombinant human vitronectin (Gibco, ThermoFisher Scientific) and passaged using enzyme-free gentle cell dissociation reagent (STEMCELL Technologies) [13]. For Protocols 2 and 3, hiPSCs were maintained on hESC-qualified Matrigel-coated (Corning) dishes in Essential 8 medium (E8) (Gibco) and passaged using Versene solution (Gibco) [37].

### Retinal organoid differentiation

The differentiation Protocol 1 [13] and Protocol 2 [38] were previously published. Protocol 3 is our novel supplemented version of Protocol 2. For all protocols, hiPSCs were expanded to reach approximately 70% confluence. At this time, defined as day 0 (D0), hiPSCs were cultured in Essential 6 medium (E6) (Gibco) for 2 days. The medium was then switched to E6 with N-2 supplement (Gibco) and changed 3 times per week. On D28, the identified NR-like structures were manually excised using a scalpel and cultured individually, or in pools of 25–30, in ultralow-attachment 24- or 6-well plates, respectively. The floating structures were cultured in DMEM/F12 + GlutaMAX (Gibco) supplemented with 1% MEM non-essential amino acids (NEAA), 1% GlutaMAX (Gibco), 2% B27 supplement (Gibco), 10 units/ml penicillin and 10 mg/ml streptomycin (Gibco). During the first week, from D28 to D35 of free-floating culture, the medium was supplemented with 10 ng/ml of animal-free recombinant human basic fibroblast growth factor (FGF2; Miltenyi Biotech). For Protocol 1, the retinal organoids were kept in the DMEM/F12 medium for long-term culture. For Protocol 2 and Protocol 3, FBS (Gibco) was added from D35. In Protocol 2, the B27 supplement was switched to B27 supplement without vitamin A (B27 -VitA) at D85. In Protocol 3, the culture medium was supplemented with 100 μM taurine at D42. At D65, the B27 supplement was switched to B27 -VitA and the medium was supplemented with 1 μM RA. At D85, the medium was supplemented with 1% N-2. RA was removed from the culture medium at D120. For all the protocols, the media was changed 2 times per week. Retinal organoids were routinely monitored using an Olympus CKX53 microscope. For each protocol, a minimum of five differentiations were performed.

### Quantitative (*q*) PCR analysis

RNA was isolated from pools of 25–30 retinal organoids, collected from two different differentiations, using the QiaShredder and RNeasy mini kit (Qiagen) according to the manufacturer’s instructions. The RNA was treated with RNase-free DNase (Qiagen), and 300 ng was reversed transcribed using the Superscript III Reverse Transcriptase Kit (Thermo Fisher Scientific). qPCR amplification was performed using 1/20 dilution of cDNA per reaction with specific primers listed in Additional file 1: Table S1. Reactions were performed using the FastStart SYBR Green I Master mix (Roche) on a LightCycler 480 II thermal cycler (Roche). The results were normalised to *GAPDH* expression levels and were analysed using LightCycler 480 software and the Microsoft Excel program. Experiments were performed in triplicate and repeated three times.

### Immunofluorescence studies

Retinal organoids were fixed in 4% paraformaldehyde (PFA; Thermo Scientific) for 20 min at 4 °C. The organoids were then washed three times in PBS and incubated in 30% sucrose in PBS at 4 °C overnight. The samples were then embedded in Tissue-Tek O.C.T. compound (Sakura) prior to snap freezing. Retinal organoids were cryosectioned in 10 μm sections, mounted onto Superfrost Plus slides (Thermo Scientific) and stored at − 80 °C. Sections were first washed with PBS before blocking and permeabilisation in PBS containing 10% donkey serum (Millipore), 5% bovine serum albumin (BSA; Sigma-Aldrich) and 0.1% Triton X-100 (Sigma-Aldrich) for 1 h at room temperature. Primary antibodies listed in Table S2 (see Additional file 1) were incubated overnight at 4 °C in PBS containing 2% donkey serum, 1% BSA and 0.1% Triton X-100. Sections were washed three times in PBS prior to incubation with fluorescence-conjugated secondary antibodies (Invitrogen-Molecular Probes) (see Additional file 1: Table S2) at 1/500 dilution for 1 h at room temperature. Hoechst 33258 solution (Sigma-Aldrich) at a final concentration of 0.2 μg/ml was also added to stain the cell nuclei. Sections processed without primary antibodies were used as negative controls. Sections were imaged using a Zeiss ApoTome 2 Upright wide-field microscope.

### Western blot analyses

Retinal organoids were lysed in RIPA buffer (Sigma-Aldrich) containing protease inhibitor cocktail tablets (Roche) and homogenised with a potter to maximise cell lysis. Samples were centrifuged at 20,000×*g* for 15 min at 4 °C, and the cleared supernatant was resuspended in 2 × Laemmli’s sample buffer (BioRad) containing 1/25 dilution of β-mercaptoethanol (Sigma-Aldrich). Samples were heated 5 min at 95 °C and immediately loaded onto an AnyKD precast MiniProtean TGX Stain Free Gel (BioRad). Proteins were transferred to a PVDF membrane using the Trans-Blot Turbo™ Transfer pack and System (BioRad). Blocking was performed in 5% skim milk in 0.5% Tween-PBS for 1 h at room temperature. Membranes were incubated with 1/1000 dilution rabbit polyclonal anti-PDE6B (ProteinTech Cat# 22063-1-AP) and 1/1000 dilution of rabbit monoclonal anti-β-tubulin (clone 9F3; Cell Signalling Cat# 2128) in blocking solution overnight at 4 °C. The membrane was washed 3 times in 0.5% Tween-PBS and incubated with 1/20,000 dilution of IRDye 800CW anti-rabbit IgG secondary antibody in blocking solution for 1 h at room temperature. The detection step was performed using a Li-COR Odyssey Imager (Li-COR Biosciences).

### Image quantification

Quantification of the brush border lengths and the lamina widths was performed using ImageJ software (https://imagej.nih.gov/ij/). Five random regions per individual retinal organoid were measured, and 29–79 organoids were analysed depending on the condition. Quantification of immunofluorescence images was performed using the Imaris Software (Bitplane). The area of fluorescence of each marker was analysed and normalised to the area of Hoechst fluorescence of the presumptive outer nuclear layer (ONL). Up to 6 regions were analysed per organoid, and 3 organoids were analysed per condition. Results were further analysed using Microsoft Excel and GraphPad Prism 8.2.1.

### Electron microscopy

Retinal organoids were washed in PHEM buffer (1X, pH 7.4) (Electron Microscopy Sciences) prior to incubation in 2.5% glutaraldehyde (Electron Microscopy Sciences) in PHEM buffer overnight at 4 °C. For transmission electron microscopy (EM), samples were washed in PHEM buffer and post-fixed in the dark at room temperature using 0.5% osmic acid for 2 h. After two washes in PHEM buffer, retinal organoids were dehydrated in a gradient series of ethanol solutions (30–100%) before being embedded in an Automated Microwave Tissue Processor for Electronic Microscopy (EmBed 812, Leica EM AMW). Thin sections (70 nm; Leica-Reichert Ultracut E) at different levels of each block were collected and counterstained with 1.5% uranyl acetate in 70% ethanol and lead citrate. Samples were observed using Tecnai F20 transmission electron microscope at 200 kV. For scanning EM, fixed samples were dehydrated using a gradient ethanol series (30–100%), followed by a 10-min incubation in ethanol–hexamethyldisilazane and then a 10-min incubation with only hexamethyldisilazane. Subsequently, samples were coated with 10 nm gold film and examined using a Hitachi S4000 and a lens detector with an acceleration voltage of 10 kV at calibrated magnifications.

### Whole organoid imaging

Whole organoids were washed once in PBS prior to fixation in 4% PFA for 45 min at 4 °C. After two washes in PBS for 20 min, the organoids were blocked and permeabilised in 10% donkey serum, 1% BSA, 0.3% Triton-X100 in PBS with gentle rocking at room temperature for 1 h. Whole organoids were then incubated with the primary antibodies (Additional file 1: Table S2) in 1% donkey serum, 1% BSA, 0.1% Triton-X100 in PBS for 4 days at 4 °C with rocking. After four washes in PBS for 20 min, organoids were incubated with the secondary antibodies (Additional file 1: Table S2) overnight at 4 °C with rocking. Organoids were washed three times in PBS for 20 min prior to incubation with 1 µg/ml DAPI (Sigma-Aldrich) for 2 h at room temperature with rocking. Two final PBS washes were performed prior to image acquisition using a Zeiss Confocal LSM880 Airyscan and a Multi-photon LSM 7MP OPO microscope.

### Statistical analyses

All data are represented as mean ± standard error of the mean (SEM). Statistical analyses were performed using GraphPad Prism software and significance was determined as *p* < 0.05, *p* < 0.01 or *p* < 0.0001. A one-way ANOVA was used to compare more than 2 groups of data, and if significant, *post hoc* analysis was performed using an unpaired Student’s t-test. In the case of small sample sizes, analyses were performed using a Mann and Whitney test and significance was determined as *p* < 0.05. The number of samples per experiment and condition are indicated in the corresponding text and figure legends.

## Results

### Culture supplements impact retinal organoid morphology

**Fig. 1:**
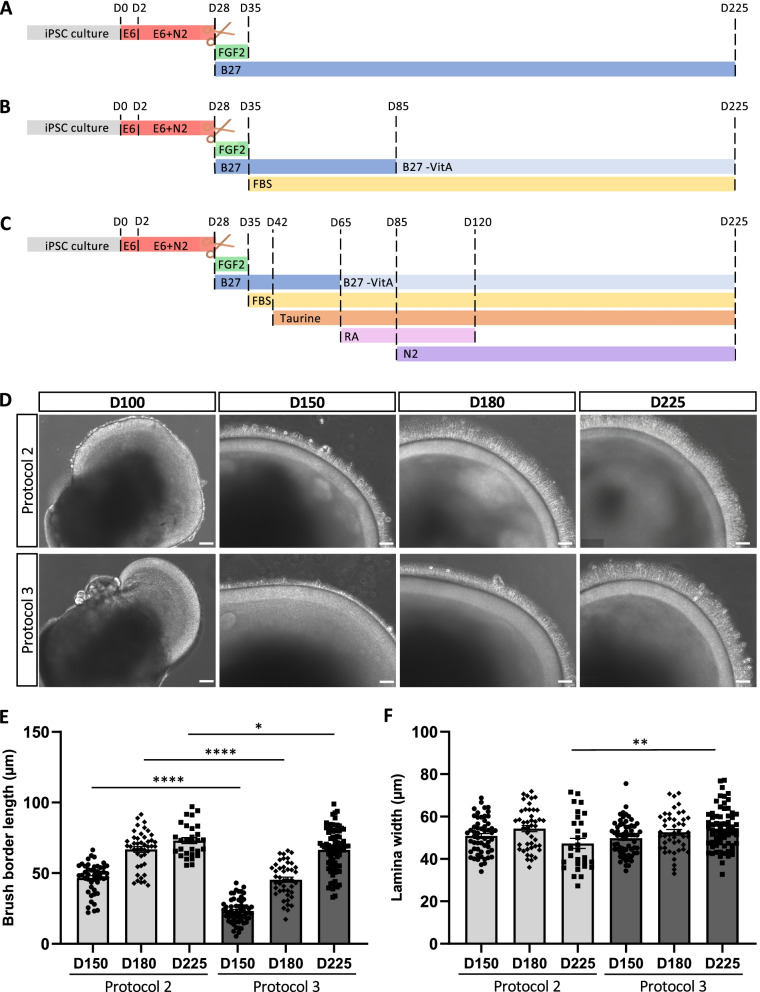
Protocol-dependent retinal organoid morphologies. Schematic timelines of the 2D–3D retinal organoid differentiation Protocols 1 (**A**), 2 (**B**) and 3 (**C**). **D** Representative bright-field images of retinal organoids at day (D) 100, D150, D180 and D225 of differentiation using Protocol 2 (upper panels) and Protocol 3 (lower panels). Scale bars = 100 μm for D100 and 50 μm for D150, D180 and D225. **E** Quantification of the length of the retinal organoid brush borders at D150, D180 and D225 for Protocol 2 (light grey bars; *n* = 47, 44 and 29 organoids, respectively) and Protocol 3 (dark grey bars; *n* = 60, 44 and 79, respectively). **F** Quantification of the width of the retinal organoid laminae at D150, D180 and D225 for Protocol 2 (light grey bars; *n* = 56, 44 and 29 organoids, respectively) and Protocol 3 (dark grey bars, *n* = 62, 46 and 67, respectively). Data are represented as mean ± SEM; *p < 0.05, **p < 0.01, ****p < 0.0001; Student’s *t* test

We compared two previously published retinal organoid differentiation protocols, referred to as Protocol 1 [10, 13] (Fig. 1A) and Protocol 2 [38] (Fig. 1B), and a novel supplemented protocol, Protocol 3 (Fig. 1C). For all protocols, differentiation was promoted by switching the hiPSC culture medium to E6, which was then supplemented with N-2 to direct anterior neural fate differentiation and promote the emergence of self-organised NR-like structures [14]. Following excision at D28, the NR-like structures were cultured transiently with FGF2 to promote proliferation and growth [16], and with B27 supplement to support long-term viability [39].

For Protocol 1, the floating NR-like structures remained in the B27-supplemented medium until maturation (Fig. 1A). As shown in Additional file 1, the Protocol 1 organoids expressed specific photoreceptor markers, such as recoverin (RCVRN) at D105 (Additional file 1: Fig. S1A), and NR2E3 (Additional file 1: Fig. S1B) and rhodopsin at D300 (Additional file 1: Fig. S1C), by immunofluorescence (IF) studies. Consistent with a previous report [10], a presumptive outer nuclear layer (ONL) did not form in these organoids, hence, marker expression was mainly concentrated in rosette-like structures. Therefore, to promote lamination of the retinal organoids, for Protocol 2 (Fig. 1B), we added FBS to the culture medium at D35 [8] and switched to B27 -VitA at D85 to enhance photoreceptor maturation [38, 40]. For Protocol 3 (Fig. 1C), we also added taurine at D42, to further promote lamination, structure and survival of the organoids [9]. Moreover, we switched to B27 -VitA at D65, at the same time as we added RA to promote photoreceptor differentiation; we removed RA at D120 to preserve photoreceptor maturation [8]. Lastly, we added N-2 at D85 to help survival of post-mitotic cells during long-term culture [39].

Both Protocols 2 and 3 gave characteristic retinal organoids with a presumptive ONL that was visible from D100 (Fig. 1D). Also at D100, we exclusively observed a nascent brush border, corresponding to the inner segments (IS) and outer segment-like (OS-like) structures of the photoreceptors, in Protocol 2 organoids; the brush border evolved and was clearly visible at D150. At D150, a nascent brush border was visible in Protocol 3 organoids (Fig. 1D). Quantitative analysis indicated that the brush border at D150 was significantly longer (*p* < 0.0001) in Protocol 2 (44.0 µm ± 2.4, *n* = 47) than Protocol 3 (23.1 µm ± 1.2, *n* = 60) (Fig. 1E) organoids. At D180, this difference remained visible (Fig. 1D) and significant (63.4 µm ± 3.4, *n* = 44, Protocol 2 *vs.* 45.3 µm ± 2.1, *n* = 44, Protocol 3; *p* < 0.0001) (Fig. 1E). In mature D225 organoids, no clear difference was visible between protocols (Fig. 1D) but quantitative analysis showed that the brush border was still significantly longer (*p* < 0.05) in Protocol 2 (72.2 µm ± 3.4, *n* = 29) than in Protocol 3 (65.9 µm ± 1.7, *n* = 79) organoids (Fig. 1E). We also measured the width of the ONL at D150, D180 and D225 (Fig. 1F). We did not observe differences between protocols until D225, when the Protocol 2 organoids showed a significantly (*p* < 0.01) thinner ONL (47.3 µm ± 2.4, *n* = 29) than the Protocol 3 organoids (54.2 µm ± 1.2, *n* = 67). This was not due to a significant difference in the overall organoid size (data not shown).

Taken together, culture supplements have an impact on organoid morphology, namely brush border formation during maturation and ONL width at maturity.

### Retinoic acid delays photoreceptor gene expression

As Protocol 1 did not give laminated organoids, we continued our studies only with Protocols 2 and 3. For these protocols, the culture conditions were identical until D42, (Fig. 1B and C), therefore, to avoid introducing additional parameters that could affect comparisons, NR-like structures collected from the same hiPSC dish were cultured in parallel without (Protocol 2) or with (Protocol 3) culture supplements until D225 (latest time point tested). We first assayed gene expression by qPCR analyses. As shown in Additional file 1, the NR-like structures collected at D35 already expressed the transcription factors *OTX2* (Additional file 1: Fig. S2A), *SIX3* (Additional file 1: Fig. S2B), *RAX* (Additional file 1: Fig. S2C) and *VSX2* (Additional file 1: Fig. S2D) involved in retinal specification, and the expression profiles were similar for both protocols.

**Fig. 2:**
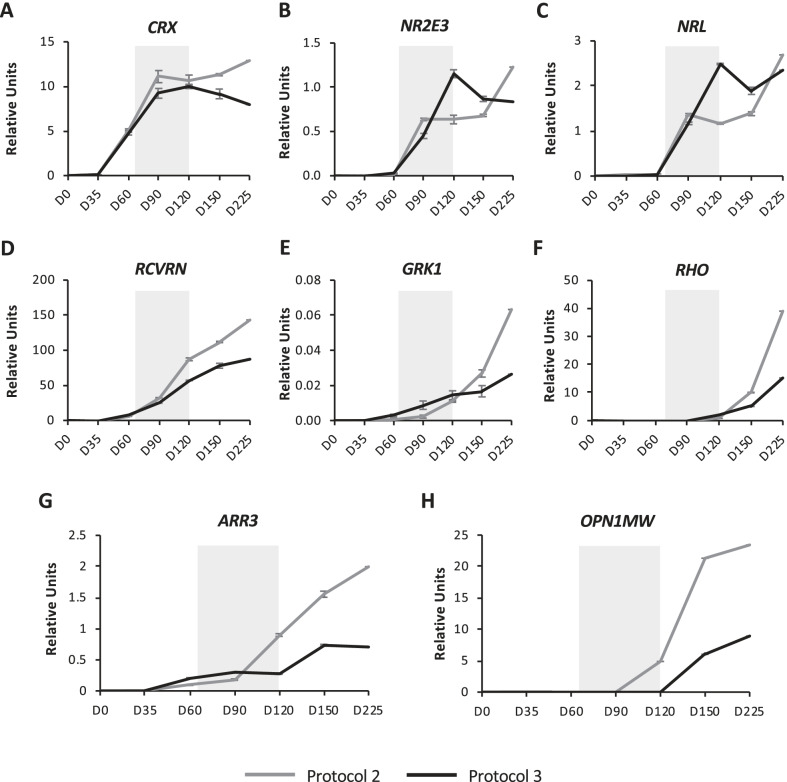
Protocol-dependent temporal expression of photoreceptor markers. Pools of 25–30 retinal organoids were collected at different differentiation time points (D35, D60, D90, D120, D150 and D225) and relative gene expression was measured by qPCR for the photoreceptor markers: *CRX* (**A**), *NR2E3* (**B**), *NRL* (**C**), *RCVRN* (**D**) *GRK1* (**E**), *RHO* (**F**) *ARR3* (**G**) and *OPN1MW* (**H**). Grey lines represent Protocol 2 and black lines Protocol 3. Light grey boxes indicate window of RA treatment for Protocol 3. Data are normalised to the housekeeping gene *GAPDH* and represented as mean ± SEM, *n* = 3

The expression profiles of the transcription factors *CRX* (Fig. 2A), *NR2E3* (Fig. 2B) and *NRL* (Fig. 2C) directing photoreceptor cell fate, were also comparable between Protocol 2 and Protocol 3 organoids, although differences were observed at specific time points. *CRX* (Fig. 2A), which drives photoreceptor differentiation, was expressed from D35 and levels increased rapidly up to D90. However, the addition of RA in Protocol 3 at D65 slowed *CRX* expression in Protocol 3 organoids compared to Protocol 2. Furthermore, at D120, when RA was removed from Protocol 3, *CRX* expression decreased in these organoids, whereas it increased in Protocol 2 organoids. The expression of *NR2E3* (Fig. 2B) and *NRL* (Fig. 2C), which encode proteins that partner with CRX to specifically drive rod differentiation, began at D60, and interestingly, for the same protocol, the expression profiles of the two genes over time were almost identical, highlighting that they are closely regulated during retinal development [41]. Furthermore, in Protocol 3 organoids, *NR2E3* and *NRL* expression continually increased from D60 to D120, *i.e.* during the RA-treatment window, consistent with previous studies showing that RA supplementation promotes rod differentiation [20]. By contrast, expression levels plateaued for Protocol 2 organoids from D90. Once RA was removed from Protocol 3 at D120, *NR2E3* and *NRL* expression levels decreased in these organoids until D150, and then remained relatively stable. By contrast, *NR2E3* and *NRL* expression levels in Protocol 2 organoids increased from D150 to D225.

The expression of the pan photoreceptor markers, recoverin (*RCVRN*; Fig. 2D) and rhodopsin kinase (*GRK1*; Fig. 2E), began between D35 and D60, and increased up to D225 in Protocol 2 and Protocol 3 organoids. However, after removal of RA from Protocol 3 at D120, *RCVRN* (Fig. 2D) and *GRK1* (Fig. 2E) expression levels remained lower in Protocol 3 organoids than in Protocol 2 organoids. Expression of the rod-specific marker rhodopsin (*RHO*) was detected from D120 in both types of organoids, but it increased sharply in Protocol 2 organoids from D150 (Fig. 2F). Interestingly, the expression of the cone-specific marker, cone arrestin-3 (*ARR3*), which began at D35 regardless of the protocol, also increased sharply from D90 to D225 in Protocol 2 organoids (Fig. 2G), whereas the addition of RA in Protocol 3 stabilised *ARR3* expression at low levels until D120. When RA was then removed, *ARR3* expression moderately increased up to D150, and then remained stable until D225. The expression of the cone-specific red–green opsin (*OPN1MW*) began at D90 for Protocol 2 organoids and continually increased until D225 (Fig. 2H). By contrast, *OPN1MW* expression was undetectable in Protocol 3 organoids until removal of RA at D120, and from then on, it was expressed at lower levels than in Protocol 2 organoids.

As shown in Additional file 1, the expression profiles of other retinal cell type markers, RGCs (*BRN3A*; Additional file 1: Fig. S2E), amacrine (*GAD2*; Additional file 1: Fig. S2F*),* bipolar (*PKCα*; Additional file 1: Fig. S2G), Müller glia (*GLAST1*; Additional file 1: Fig. S2H) and horizontal (*LIM1*; Additional file 1: Fig. S2I) cells, were comparable between protocols although *BRN3A*, *GAD2* and *PKCα* reached higher levels in Protocol 2 organoids, and *GLAST1* and *LIM1* in Protocol 3 organoids. By D225, the markers were expressed at similar levels in both types of organoids. Furthermore, regardless of protocol, *BRN3A* expression was lost from D120, in accordance with previous studies reporting the loss of RGCs in mature organoids [11, 14, 33].

Taken together, comparable expression profiles are observed in organoids generated using non-supplemented or supplemented culture conditions; however, under supplemented conditions, the presence of RA appears to restrain cone fate differentiation.

### Supplemented media promotes rod-rich organoids and preserves ONL integrity

**Fig. 3:**
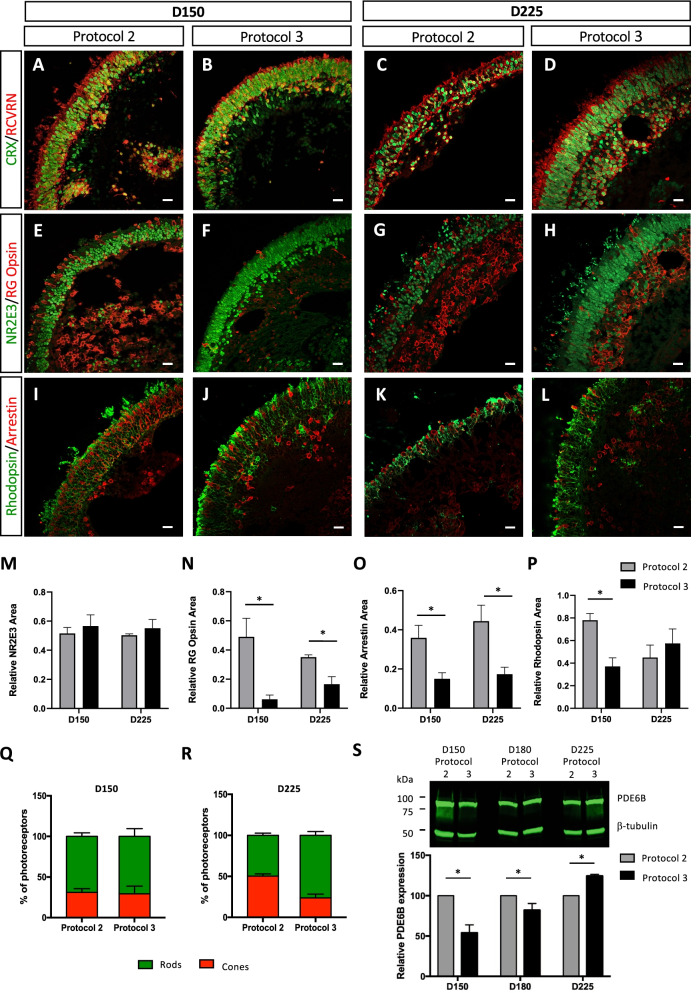
Qualitative and quantitative analysis of photoreceptor markers in mid-stage and mature organoids. Retinal organoids were analysed at D150 (**A**, **B**, **E**, **F**, **I**, **J**) and D225 (**C**, **D**, **G**, **H**, **K**, **L**) of differentiation. Representative images of the expression of the nuclear cone–rod homeobox protein (CRX; in green) and the calcium binding protein recoverin (RCVRN; in red) in Protocol 2 (**A**, **C**) and Protocol 3 (**B**, **D**) organoids. Representative images of the expression of the rod transcription factor NR2E3 (in green) and the cone-specific red–green opsins (RG opsin; in red) in Protocol 2 (**E**, **G**) and Protocol 3 (**F**, **H**) organoids. Representative images of rod-specific rhodopsin (in green) and cone-specific arrestin (in red) expression in Protocol 2 (**I**, **K**) and Protocol 3 (**J**, **L**) organoids. Scale bars = 20 μm. Quantification analysis of the relative areas of NR2E3 (**M**), RG opsin (**N**), arrestin (**O**) and rhodopsin (**P**) fluorescence within the ONL normalised to the area of Hoechst fluorescence in the ONL (see Additional file 1: Fig. S3). Quantification was performed on 6–13 images per organoid and 3 organoids were analysed per condition. Data are represented as mean ± SEM; *p < 0.05, *n* = 3; Mann and Whitney test. Quantification of rods (green bars) and cones (red bars), as determined by areas of normalised rhodopsin and arrestin fluorescence, respectively, and expressed as a percentage of the total photoreceptors at D150 (**Q**) and D225 (**R**) in Protocol 2 and Protocol 3 organoids. **S** Representative western blot (upper panel) of PDE6B expression in Protocol 2 and Protocol 3 organoids collected at D150, D180 and D225 of differentiation. β-tubulin was used as a loading control. Quantification (lower panel) of PDE6B expression normalised to β-tubulin expression of two independent experiments. For each time point, PDE6B levels in Protocol 3 organoids are expressed relative to those in Protocol 2 organoids. Data are represented as mean ± SEM; *p < 0.05; Mann and Whitney test

Due to the differences observed in the brush border length, ONL width and gene expression profiles between Protocol 2 and Protocol 3 organoids, we next analysed common photoreceptor-specific markers by IF studies at mid- (D150) and mature (D225) stages of differentiation (Fig. 3). CRX was mainly restricted to the nuclei of the photoreceptors, and at D150, the ONL of the Protocol 2 organoids (Fig. 3A) was less tightly packed than that of Protocol 3 organoids (Fig. 3B). This difference was even more pronounced at D225 when the ONL was visibly thinner in Protocol 2 organoids (Fig. 3C and D), consistent with quantification of bright-field images (Fig. 1F). RCVRN was expressed throughout the length of the photoreceptors, and at D150, RCVRN expression extended further from the ONL in Protocol 2 (Fig. 3A) than in Protocol 3 (Fig. 3B) organoids. This was in accordance with the longer brush border observed by bright-field microscopy (Fig. 1D). At D225, RCVRN expression in Protocol 3 organoids extended further from the ONL than at D150 (Fig. 3D), indicative of the prolongation of the IS and OS-like structures and consistent with the longer brush border measurements at this time point (Fig. 1E). However, at D225 the deterioration of the ONL in Protocol 2 organoids (Fig. 3C) did not allow the comparison of the length of the IS and OS-like structures by RCVRN expression with that of the Protocol 3 organoids (Fig. 3D).

Similar to the previous CRX results, NR2E3 was expressed in photoreceptor nuclei and the ONL appeared less tightly packed in Protocol 2 organoids at D150 (Fig. 3E), compared to Protocol 3 organoids (Fig. 3F), and this difference was even more flagrant at D225 (Fig. 3G and H). Quantification of the area of NR2E3 fluorescence, relative to Hoechst fluorescence in the ONL shown in Additional File 1: Fig. S3, showed no significant differences between the two types of organoids (Fig. 3M). We detected expression of the cone visual pigment red–green opsin (RG opsin) at D150 (Fig. 3E and F) and D225 (Fig. 3G and H) for both protocols and clearly observed more RG opsin-positive cells in Protocol 2 organoids (Fig. 3E and G), as compared to Protocol 3 organoids (Fig. 3F and H). Consistently, quantification of the relative area of RG opsin fluorescence within the ONL showed that it was significantly higher (*p* < 0.5; *n* = 3) for Protocol 2, compared to Protocol 3, organoids at both time points (eightfold at D150 and twofold at D225; Fig. 3N).

In accordance with the RG opsin results, we observed more cone arrestin-positive cells in the ONL of the Protocol 2 organoids at D150 (Fig. 3I) and D225 (Fig. 3K), compared to Protocol 3 organoids (Fig. 3J and L). This increase was confirmed to be statistically different (*p* < 0.5; *n* = 3) by quantification of the relative area of arrestin fluorescence relative to the Hoechst fluorescence (Additional file 1: Fig. S3) in the ONL (twofold at D150 and threefold at D225; Fig. 3O). Rhodopsin expression was abundant in both protocols and detected throughout the length of the photoreceptors at D150 (Figs. 3I and J) and D225 (Fig. 3K and L). However, at D225, the rhodopsin-positive cells for Protocol 3 organoids (Fig. 3L) appeared longer than those for Protocol 2 (Fig. 3K). Interestingly, the quantification of the area of rhodopsin fluorescence, relative to Hoechst fluorescence in the ONL shown in Additional File 1: Fig. S3, suggested that Protocol 2 had significantly twofold higher (*p* < 0.5; *n* = 3) rhodopsin levels at D150, whereas Protocol 3 organoids showed a tendency towards higher expression at D225 (Fig. 3P).

Lastly, we analysed the percentage of rods and cones in organoids for each protocol, and at D150, rods predominated (68.9% ± 2.58% Protocol 2 and 70.8% ± 5.49% Protocol 3) as compared to cones (31.1% ± 2.58% Protocol 2 and 29.3% ± 5.49% Protocol 3) regardless of the protocol used (Fig. 3Q). By contrast, at D225, the percentage of cones (50.3% ± 1.54%) in Protocol 2 organoids had increased to become equivalent to that of rods (49.7% ± 1.54%). Conversely, the percentage of cones (23.8% ± 2.70%) in Protocol 3 organoids had decreased, rendering the rods clearly predominant (76.2% + 2.70%) (Fig. 3R). We further confirmed the changing rod population over time semi-quantitatively using western blot analysis of another mature rod marker, PDE6B (Fig. 3S). At D150, PDE6B expression was significantly twofold higher (*p* < 0.5; *n* = 2) in Protocol 2, than Protocol 3, organoids. At D180, PDE6B expression levels in Protocol 2 organoids were closer to those of Protocol 3 but still significantly 1.2-fold higher (*p* < 0.5; *n* = 2). However, at D225, the situation had reversed and Protocol 3 organoids showed significantly 1.2-fold higher PDE6B expression levels (*p* < 0.5; *n* = 2) than Protocol 2 organoids. These data confirm the earlier differentiation of Protocol 2 organoids and the predominant rod population in mature Protocol 3 organoids.

**Fig. 4:**
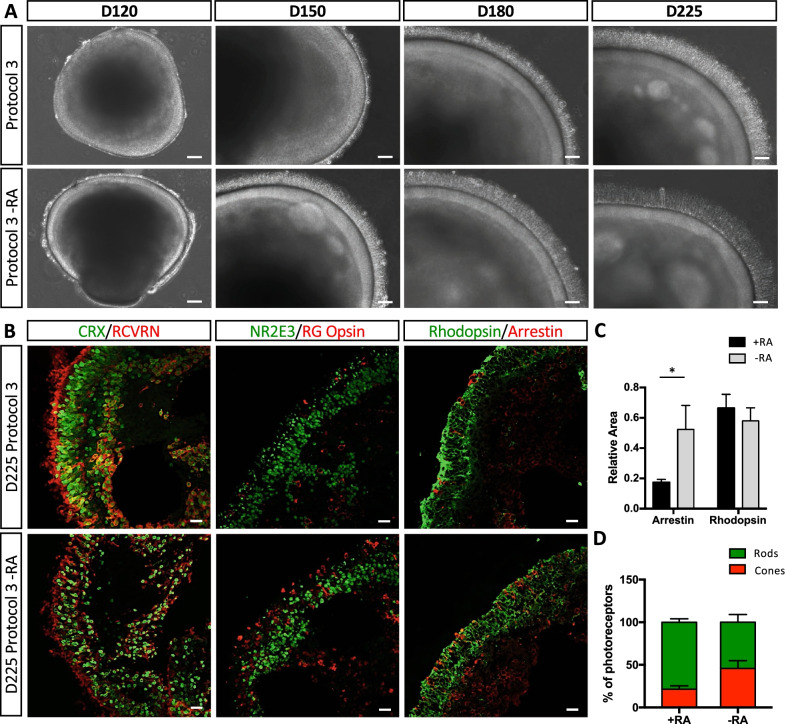
Qualitative and quantitative analysis of photoreceptor markers in the absence of RA. **A** Representative bright-field images of retinal organoids at D120, D150, D180 and D225 of differentiation using Protocol 3 (upper panels) or Protocol 3 without RA (Protocol 3 -RA; lower panels). Scale bars = 100 μm for D120 and 50 μm for D150, D180 and D225. **B** Representative IF images of D225 organoids generated using Protocol 3 (upper panels) or Protocol 3 -RA (lower panels): Left-hand panels, CRX (in green) and RCVRN (in red); middle panels, NR2E3 (in green) and RG opsin (in red); right-hand panels, rhodopsin (in green) and arrestin (in red). Scale bars = 20 μm. **C** Quantification analysis of the relative areas of arrestin and rhodopsin fluorescence within the ONL normalised to the area of Hoechst fluorescence in the ONL (see Additional file 1: Fig. S4). Quantification was performed on 3–5 images per organoid and 3 organoids were analysed per condition. Data are represented as mean ± SEM; *p < 0.05, *n* = 3; Mann and Whitney test. **D** Quantification of rods (green bar) and cones (red bar), as determined by areas of normalised rhodopsin and arrestin fluorescence, respectively, and expressed as a percentage of the total photoreceptors at D225 in Protocol 3 or Protocol 3 -RA organoids

The qPCR results suggested that RA plays a pivotal role in photoreceptor gene expression. Therefore, to assay whether the observed morphological changes were exclusively due to the presence of RA, we differentiated in parallel retinal organoids under Protocol 3 conditions with or without RA (Protocol 3 -RA). Similar to our observations in Protocol 2 organoids, we detected a brush border earlier in Protocol 3 -RA organoids that evolved more rapidly than that of Protocol 3 organoids (Fig. 4A). At D225, IF studies to assess CRX and RCVRN expression detected a less tightly packed ONL in the Protocol 3 -RA organoids as compared to Protocol 3 organoids (Fig. 4B). In addition, Protocol 3 -RA organoids contained more cones as determined by RG opsin and arrestin expression (Fig. 4B), compared to Protocol 3 organoids. Quantification of the area of arrestin fluorescence, relative to Hoechst fluorescence in the ONL shown in Additional file 1 (Fig. S4), confirmed a significant threefold difference (*p* < 0.5; *n* = 3; Fig. 4C). No significant differences in rhodopsin expression were observed for Protocol 3 and Protocol 3 -RA organoids (Fig. 4B and C), although Protocol 3 organoids showed a tendency towards higher levels. These data are reminiscent of the observations at D225 for Protocol 2 and Protocol 3 organoids (Fig. 3P). Lastly, we analysed the percentage of rods and cones and detected a higher percentage of cones (45.7% ± 9.17%) in Protocol 3 -RA organoids as compared to Protocol 3 organoids (21.4% ± 4.02%) (Fig. 4D). Conversely Protocol 3 organoids had a higher percentage of rods (78.6% ± 4.02%) compared to Protocol 3 -RA organoids (54.3, ± 9.17%). Notably, the cone and rod percentages of the D225 Protocol 3 -RA organoids were almost identical to those of Protocol 2 organoids.

In conclusion, under non-supplemented conditions, and specifically in the absence of RA, the ONL of the retinal organoids is less tightly packed and the photoreceptors appeared shorter in comparison to supplemented conditions. In addition, the absence of RA results in cone-rich organoids, whereas the presence of RA result in rod-rich organoids.

### Supplemented media improves the structure of the photoreceptor layer

**Fig. 5:**
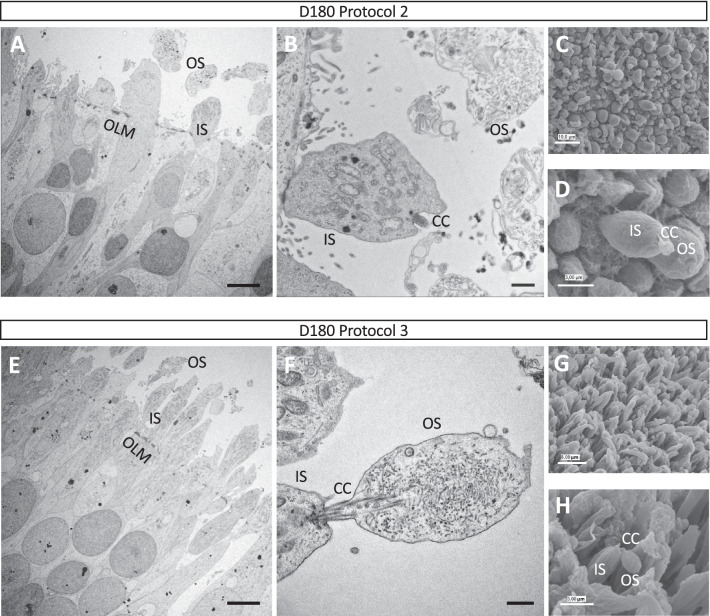
Ultrastructure and surface analysis of retinal organoids. Representative images of transmission EM analysis of D180 Protocol 2 (**A**) and Protocol 3 (**E**) organoids showing the ultrastructure of the ONL. The outer limiting membrane (OLM), the inner segments (IS) and the outer segment (OS)-like structures of the photoreceptors are indicated. Scale bars = 5 μm. Higher magnification images of Protocol 2 (**B**) and Protocol 3 (**F**) organoids showing in more detail the ultrastructure of the IS and OS-like structures of the photoreceptors, as well as the connecting cilium (CC). Scale bars = 1 μm (B) and 500 nm (F). Representative scanning EM images of Protocol 2 (**C**) and Protocol 3 (**G**) organoids showing the differential cellular shape of the predominant cones and rods, respectively. Higher magnification images showing the IS, CC and OS-like structures of photoreceptors for Protocol 2 (**D**) and Protocol 3 (**H**). Scale bars = 10 μm (C), 3 μm (D), 8 µm (G) and 3 µm (H)

We next assayed the ultrastructure of the ONL and the photoreceptors of the retinal organoids using transmission electron microscopy (EM) at D180 (Fig. 5). As suggested by the IF studies, the nuclei and the cell bodies of the photoreceptors were less organised in Protocol 2 organoids (Fig. 5A), as compared to their perfect alignment forming a highly structured ONL in Protocol 3 organoids (Fig. 5E). Despite this organisational difference, we observed characteristic features of mature photoreceptors for both Protocol 2 and 3 organoids, such as the outer limiting membrane (OLM) and the connecting cilium (CC) linking IS and OS-like structures that contained rudimentary photoreceptor discs (Fig. 5B and F). In parallel we evaluated the retinal organoid brush border using scanning EM, which revealed differences in photoreceptor shape between protocols (Fig. 5C and G). The photoreceptors of Protocol 2 organoids were larger, rounder and shorter, consistent with the shape of cones (Fig. 5C), whereas the photoreceptors of Protocol 3 organoids were thinner and longer, consistent with the shape of rods (Fig. 5G). Similar to the transmission EM results, we observed the CC and the OS-like structures of the photoreceptors on the surface of both Protocol 2 and 3 organoids (Figs. 5D and H).

Taken together, under supplemented conditions, the photoreceptor layer of retinal organoids is more highly organised compared to non-supplemented conditions. In addition, the cone-rich (non-supplemented conditions) and rod-rich (supplemented conditions) organoids can be distinguished by their surface morphology.

### Supplemented media promotes stratification and elongation of the photoreceptors

**Fig. 6:**
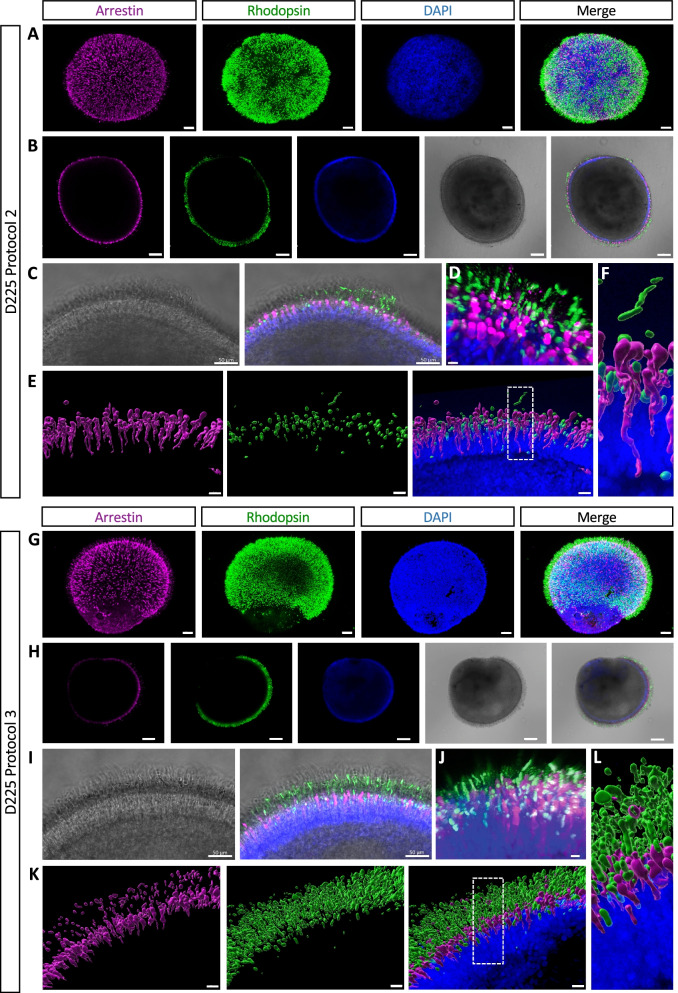
Three-dimensional imaging of mature retinal organoids. **A** Confocal imaging of D225 Protocol 2 organoids showing the arrestin-stained cones (in purple) and rhodopsin-stained rods (in green); DAPI-stained nuclei (in blue). Scale bars = 100 µm. **B** A single confocal plane showing the cones (in purple), rods (in green) and nuclei (in blue), the bright-field image, and a merge of the four channels. Scale bars = 150 µm. **C** Higher magnification of the bright-field and merged images in (**B**). Scale bars = 50 µm. **D** High resolution Aryscan imaging of the organoid surface showing the rods (in green) and cones (in purple); nuclei stained in blue. Scale bar = 10 µm. **E** Biphoton imaging and 3D reconstruction showing the cones (in purple), the OS-like segments of the rods (in green) and the merge of the two channels with the DAPI-stained ONL (in blue). Scale bars = 20 µm. Enlarged boxed area shown in (**F**). **G** Confocal imaging of Protocol 3 organoids showing cones (in purple) and rods (in green); DAPI-stained nuclei (in blue). Scale bars = 100 µm. **H** A single confocal plane showing the cones (in purple), rods (in green) and nuclei (in blue), the bright-field image and a merge of the four channels. Scale bars = 150 µm. **I** Higher magnification of the bright-field and merged images in (**H**). Scale bars = 50 µm. **J** High resolution Aryscan imaging showing the rods (in green) and cones (in purple); nuclei stained in blue. Scale bar = 10 µm. **E** Biphoton imaging and 3D reconstruction showing the cones (in purple), rods (in green) and the merge of the two with the DAPI-stained ONL (in blue). Scale bars = 20 µm Enlarged boxed area shown in (**L**)

To better characterise the differential shape and spatial distribution of the photoreceptors at the surface of the retinal organoids observed by scanning EM, we performed IF studies on whole mature organoids (D225) using arrestin and rhodopsin as markers of cone and rod photoreceptors, respectively. Confocal analysis at low magnification of the surface of Protocol 2 organoids showed a well-distinguished, regular distribution of arrestin-positive cells, and intensely stained but irregularly distributed rhodopsin-positive cells (Fig. 6A). Analysis of a single confocal plane confirmed the regular layer (in distribution and width) of cones, in contrast to the irregular layer of rods, around the organoid (Fig. 6B). The DAPI-stained nuclei clearly formed a continuous layer on the inner side of the cones, whereas the rhodopsin signal was either interspersed among the cones or extended beyond them in patches. Higher magnification showed that within these patches, the rods extended the width of the brush border (Fig. 6C). Moreover, high resolution confocal microscopy using the Airyscan module confirmed that the rod OS-like structures had a distinctly thin and elongated shape, whereas the cones were more rounded (Fig. 6D), consistent with the scanning EM images. To achieve a better depth perception without the need for clearing, we analysed the retinal organoids by biphoton microscopy and Imaris 3D reconstruction, which confirmed a well-defined cone layer with interspersed rods, and the occasional rod OS-like structure extending past the cones (Fig. 6E and F), as well as the differential shape between rods and cones.

In stark contrast to Protocol 2 organoids, low magnification confocal analysis of the surface of Protocol 3 organoids showed a regular distribution of cones, as well as intensely stained and regularly distributed rods (Fig. 6G), which was confirmed by single plane analysis (Fig. 6H). At higher magnification, both cones and rods appeared longer than those observed in Protocol 2 organoids (Fig. 6C), and the rods consistently extended beyond cones the full width of the brush border (Fig. 6I). This was further confirmed using high resolution microscopy (Fig. 6J). Biphoton microscopy and Imaris 3D reconstruction confirmed a dense layer of rods, which were evenly distributed and extended beyond the cones (Fig. 6K), and the elongated rod OS-like structures clearly contrasted with the shorter bulbous cone structures (Fig. 6L).

Taken together, organoids cultured under supplemented conditions showed a regular and stratified distribution of photoreceptors, with longer cone and rod OS-like structures, as compared to organoids cultured under non-supplemented conditions. In both cases, rod and cone photoreceptors could be distinguished by their distinct shapes.

## Discussion

The generation of reliable and homogenous human retinal models to better model IRDs and to test promising therapies has been a challenge for eye research. Since the publication of the first 3D retinal organoid protocols [8–10], multiple modifications have been proposed, and to date, there is not one gold standard protocol used by all laboratories. It is hence difficult to draw conclusions concerning the optimal protocol and to compare organoids generated from different hiPSC lines and/or laboratories. Therefore, in this study, we directly investigated the effect of media supplements on the morphology of retinal organoids differentiated from the same hiPSC line, a comparison that was still missing in the literature. Furthermore, this parallel comparison allowed us to investigate if the time-consuming and labour-intensive work of coordinating various media at different culture times has a direct impact on photoreceptor differentiation.

We designed a novel retinal organoid differentiation protocol to study the effect of the most commonly used media supplements, such as taurine, RA and N-2. Unexpectedly, our results indicate that supplemented media delays photoreceptor differentiation at early stages but highly improves overall structure and stratification of the mature retinal organoid. We confirm that the addition of FBS promotes lamination of retinal organoids, as previously described [9]. A direct effect of taurine or N-2 on the expression of photoreceptor-specific markers, as analysed by qPCR, was not observed. By contrast, the effect of RA was clearly visible by direct modulation of the rod photoreceptor cell-fate genes *NRL* and *NR2E3*. This is in accordance with previous studies showing that RA promotes photoreceptor development and retinogenesis [42, 43], enhances expression of *NRL* [44] and drives rod differentiation [20]. Thus, of the commonly used media supplements, only RA has a measurable impact on retinal differentiation.

Furthermore, we show for the first time that the effect of RA supplementation can be directly evaluated at the surface of retinal organoids. Interestingly, without RA and regardless of the presence or absence of taurine and N-2, the brush border of the organoids appears as early as D100, which was not the case if RA was present. Furthermore, the higher expression levels of the cone markers *ARR3* and *OPN1MW*, and the lack of detectable *RHO* expression at D100, suggest that this early brush border is composed of cone photoreceptors. This was further confirmed by preliminary IF studies on D100 organoids, which showed 1) a distinct CRX-positive and NRL-negative outer layer of photoreceptor nuclei and 2) the presence of arrestin- and RG opsin-positive cells in the ONL exclusively under non-supplemented conditions (Additional file 1: Fig. S5). Taken together, these data strongly support the notion that the difference in surface morphology is primarily due to RA supplementation, which we show restrains cone differentiation. This is consistent with a study in mouse retinal organoids showing that RA signalling regulates cone maturation by suppressing arrestin expression (45).

In addition to the earlier photoreceptor differentiation in the absence of RA and regardless of other supplements, clear differences were observed regarding photoreceptor structure and population in mature retinal organoids. Consistent with the early development of the cone-rich brush border, mature organoids differentiated without RA had a predominance of cones at their surface. This is compatible with the relatively high levels of *ARR3* and *OPN1MW* expression at D225 and the cone fluorescence area quantified from the IF studies. When expressed as a percentage of total photoreceptors, the proportion of rods and cones in the organoids cultured without RA appeared equal. It is possible that the longer length of the rods compared to cones could influence the fluorescence area quantifications leading to an underestimation of the cone population. Conversely, the organoids differentiated with RA had a regular and dense population of rods at the surface. This is consistent with the quantification of the cone and rod proportions at D225, which clearly demonstrate a predominant rod population in RA-cultured organoids. Surprisingly, we observed higher *RHO* expression levels at D225 for organoids cultured without RA as compared to RA-cultured organoids. The reason for this discrepancy is currently unclear but it may reflect a heterogeneity in the maturation of the rod OS in the organoids pooled for qPCR studies. Nevertheless, the higher PDE6B expression observed by western blot analyses, as well as the higher degree of ONL organisation observed by transmission EM studies, in the RA organoids argue in favour of the IF observations. Hence, overall, the data confirm the role of RA in rod development but, more importantly, show for the first time that RA delays the initial stages of photoreceptor differentiation to result in a more highly structured photoreceptor layer at maturity.

Such highly structured retinal organoids represent more pertinent models for studying IRDs and for the development of novel therapies [46]. Furthermore, depending on the IRD of interest, retinal organoids must recapitulate to some extent the ratio of cones to rods of the human retina. The cone–rod ratio of the mature organoids cultured in the presence of RA was 1:3, which is similar to that described for the parafoveal region of the human retina [47]. By contrast, the 1:1 cone–rod ratio of organoids cultured in the absence of RA would represent a region closer to the foveal centre. Consistently, Kim and colleagues reported a differentiation protocol comprising FBS and taurine but not RA that resulted in cone-rich retinal organoids with a ratio of 1.4:1 [48]. Regrettably, the overall structure and correct stratification of the mature cone-rich retinal organoid was not evaluated. Nonetheless, our results suggest that cone-rich organoids generated in the absence of RA could be a useful model for studying IRDs affecting the macula, if analyses are performed at mid-stages of differentiation prior to the observed loss of ONL integrity. The question remains as to why the cone-rich organoids lose their integrity over time and whether this could be due to the low percentage of rods. A parallel could be made with the natural history of the IRD retinitis pigmentosa, which is characterised by an initial loss of rods that leads to a secondary loss of cones [49]. In addition, it has been shown that rods secrete neurotrophic factors that contribute to cone survival, such as Rod-derived Cone Viability Factor (RdCVF), and that if used as a therapeutic agent, this factor can prevent cone degeneration in RP [50]. Therefore, it is tempting to speculate that it may be important to maintain a certain population of rods in the retinal organoids to prevent photoreceptor degeneration over long-term culture.

The loss of ONL integrity in mature organoids cultured without RA, raises consideration for translational medicine applications such as cell transplantation, which is an appealing treatment option for late-stage IRD patients. Retinal organoids have already been used for pre-clinical photoreceptor transplantation efforts [14, 16, 18, 31], whereby post-mitotic precursors are collected between D100 and D120, i.e. during the RA-treatment window. Our results suggest that transplantable populations may differentiate better in the long term if cultured with RA. Encouragingly, RA supplementation also seems to improve the elongation of the OS-like structures of the photoreceptors, although these still contain only rudimentary discs. Future directions for improving retinal organoids should include protocols leading to OS with stacked membrane discs. Given the crucial role of the RPE in photoreceptor maturation and OS homeostasis [51], a step in this direction would be the use of organ-on-a-chip models to co-culture the developing organoid in the presence of RPE [52]. In addition, microfluidic technologies could be used to vascularise the retinal organoid [53], which would fully mimic the microenvironment of the human retina. Lastly, a particularly noteworthy study recently reported 3D brain organoids containing bilaterally symmetric optic vesicles with corneal and lens-like cells, as well as retinal progenitor cells (RPCs) and RPE (54). Although these structures were only viable for short culture times (D60), further development of such eye–brain organoids would have high potential for understanding and treating eye development and early retinal diseases.

## Conclusions

In summary, we have demonstrated that RA supplementation delays initial photoreceptor differentiation to highly improve the overall structure and stratification of mature retinal organoids. Furthermore, RA modulates photoreceptor populations to give rod-rich organoids, which can be readily evaluated by surface imaging. These results suggest that RA supplementation has implications for both disease modelling and generating transplantable cell populations.

## Supplementary Information

**Additional file 1**. Supplementary Information containing details of primers and antibodies (Tables S1 and S2) used in the study, and the Figures showing the characterisation of Protocol 1 organoids (S1), the qPCR analysis of the expression of early retinal specification markers and non-photoreceptor markers in Protocol 2 and 3 organoids (S2), the Hoechst staining of the organoids  shown in Figs. 3 and 4 (S3 and S4), and the morphology and staining of early-stage organoids (S5).

## Data Availability

All data generated or analysed during this study are included in this published article and its supplementary information files.

## Abbreviations

ARR3: Cone arrestin-3
BSA: Bovine serum albumin
CC: Connecting cilium
CRX: Cone–rod homeobox
E8: Essential 8 medium
E6: Essential 6 medium
EM: Electron microscopy
FBS: Foetal bovine serum
FGF2: Basic fibroblast growth factor
GRK1: G protein-coupled receptor kinase 1
hiPSC: Human-induced pluripotent stem cell
IF: Immunofluorescence
IRDs: Inherited retinal dystrophies
IS: Inner segment
NEAA: Non-essential amino acids
NR2E3: Nuclear receptor subfamily 2 group E member
NRL: Neural retina leucine zipper
NR: Neural retina
OLM: Outer limiting membrane
ONL: Outer nuclear layer
OS: Outer segment
PFA: Paraformaldehyde
RA: Retinoic acid
RCVRN: Recoverin
RGCs: Retinal ganglion cells
RHO: Rhodopsin
RG opsin: Red–green opsin
RPE: Retinal pigment epithelium
SEM: Standard error of the mean
