# Supplementary material for: Retinoic acid delays initial photoreceptor differentiation and results in a highly structured mature retinal organoid
Source: Stem Cell Res Ther. 2022 Sep 16;13:478. doi: 10.1186/s13287-022-03146-x (PMC9482314; doi:10.1186/s13287-022-03146-x)
Supplement: Supplementary file 1 — Additional file 1. Supplementary Information containing details of primers and antibodies (Tables S1 and S2) used in the study, and the Figures showing the characterisation of Protocol 1 organoids (S1), the qPCR analysis of the expression of early retinal specification markers and non-photoreceptor markers in Protocol 2 and 3 organoids (S2), the Hoechst staining of the organoids shown in Figs. 3 and 4 (S3 and S4), and the morphology and staining of early-stage organoids (S5). [file 13287_2022_3146_MOESM1_ESM.pdf]

# Additional File 1

## Retinoic acid delays initial photoreceptor differentiation and results in a highly structured mature retinal organoid

Carla Sanjurjo-Soriano, Nejla Erkilic, Krishna Damodar, Hassan Boukhaddaoui, Michalitsa Diakatou, Marcela Garita-Hernandez, Daria Mamaeva, Gregor Dubois, Zhour Jazouli, Carla Jimenez-Medina, Olivier Goureau, Isabelle Meunier & Vasiliki Kalatzis

**Table S1:** Primers used for qPCR analysis of gene expression

**Table S2:** Primary and secondary antibodies used for immunofluorescence studies

**Figure S1:** Expression of photoreceptor markers in non-laminated Protocol 1 organoids

**Figure S2:** Temporal gene expression of eye-field specification and non-photoreceptor markers in Protocol 2 and Protocol 3 organoids

**Figure S3:** Immunofluorescence studies of Protocol 2 and Protocol 3 organoids

**Figure S4:** Immunofluorescence studies of Protocol 3 organoids with or without RA

**Figure S5:** Cones in early-stage Protocol 2 and Protocol 3 organoids

**Table S1:** Primers used for qPCR analysis of gene expression

| Gene | Forward primer 5'-3' | Reverse primer 5'-3' | Amplicon | Efficiency |
|---|---|---|---|---|
| *ARR3* | CCCAGAGCTTTGCAGTAACC | CACAGGACACCATCAGGTTG | 189 bp | 1.79 |
| *BRN3A* | AGAAGCAGAAGCGGATGAAA | CAGAGAATGGGTGGAGGAAA | 194 bp | 1.90 |
| *CRX* | TGATGGTGGGATTGGAAAAT | GCCTTGTGAACTCCACCAAT | 248 bp | 2.04 |
| *GAD2* | TCGTCAGATTCCAAGTGCTG | TAGGGCATTTCTACCCGTTG | 169 bp | 1.81 |
| *GLAST1* | AACCCATCGACAGTGAAACC | TCCCTTGTGTTTTGGAGGAC | 184 bp | 1.93 |
| *LIM1* | GCCAAAGAGAACAGCCTTCACTC | GGTCGTCATTCTCGTTGCTACC | 148 bp | 1.94 |
| *NRL* | GGCTCCACACCTTACAGCTC | ATGGCCTCTTCAGGACTCAG | 170 bp | 1.96 |
| *NR2E3* | CACTTCATGGCCAGCCTTAT | CTGGAGAACACAGGCAGGTT | 215 bp | 1.80 |
| *OPN1MW* | GAACCAGGTCTATGGCTACTTCG | TCTCACATTGCCAAAGGGCTT | 154 bp | 2.04 |
| *OTX2* | GCAGAGGTCCTATCCCATGA | CTGGGTGGAAAGAGAAGCTG | 211 bp | 1.95 |
| *PKC*$\alpha$ | GTGGCAAAGGAGCAGAGAAC | TGTAAGATGGGGTGCACAAA | 151 bp | 1.92 |
| *RAX* | GTCCCTAAGCGTGCTTTCAG | CATGCCAGGGTCTTGGTACT | 200 bp | 1.99 |
| *RCVRN* | AGCTCCTTCCAGACGATGAA | CAAACTGGATCAGTCGCAGA | 150 bp | 2.02 |
| *RHO* | ACAGGATGCAATTTGGAGGGC | GCTCATGGGCTTACACACCA | 111 bp | 2.06 |
| *GRK1* | GCCCGTGAAGTACCCTGATA | TTGGAGTCTGGGATGAAAGG | 198 bp | 2.02 |
| *SIX3* | CAGAAGACGCATTGCTTCAA | GCCGGTTCTTAAACCAGTTG | 151 bp | 2.00 |
| *VSX2* | CTGACTCTGGACCATGCTGA | GAGCTGGGAAGGAGGACTCT | 189 bp | 1.87 |
| *GAPDH* | AACCATGAGAAGTATGACAAC | CTTCCACGATACCAAAGTT | 112 bp | 2.02 |

**Table S2:** Primary and secondary antibodies used for immunofluorescence studies

| **Antibody** | **Dilution** | **Company Cat #; RRID** |
|---|---|---|
| Goat polyclonal anti-Arrestin 3 | 1/200 | Novus Biologicals Cat# NBP1-37003; AB_2060085 |
| Mouse monoclonal anti-CRX, clone 4G11 | 1/2000 | Abnova Cat# H00001406-M02;AB_606098 |
| Mouse monoclonal anti-NR2E3/PNR, clone H7223 | 1/150 | R&D Systems Cat# PP-H7223-00; AB_2155481 |
| Rabbit polyclonal anti-Opsin, Red/Green | 1/500 | Millipore Cat# AB5405; AB_177456 |
| Rabbit polyclonal anti-Recoverin | 1/2000 | Millipore Cat# AB5585; AB_2253622 |
| Mouse monoclonal anti-Rhodopsin, clone 4D2 | 1/250 | Millipore Cat# MABN15; AB_10807045 |
| Donkey anti-Mouse IgG (H+L), Alexa Fluor 488 | 1/500 | ThermoFisher Scientific Cat# A-21202; AB_141607 |
| Donkey anti-Rabbit IgG (H+L), Alexa Fluor 594 | 1/500 | ThermoFisher Scientific Cat# A-21207; AB_141637 |
| Donkey anti-Goat IgG (H+L), Alexa Fluor 594 | 1/500 | ThermoFisher Scientific Cat# A-11058; AB_2534105 |

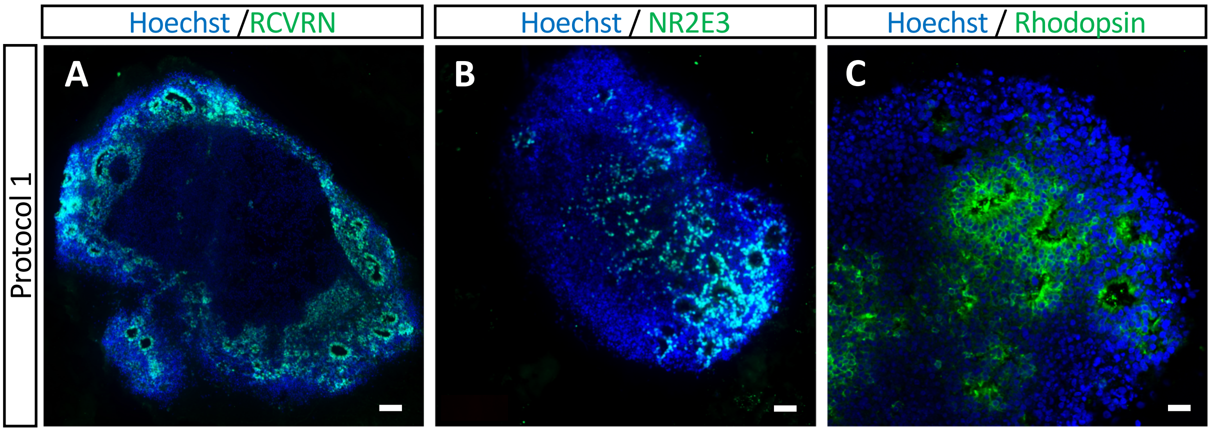


**Figure S1: Expression of photoreceptor markers in non-laminated Protocol 1 organoids. A**) Representative image of RCVRN expression in green, which is localized to rosette-like structures of the non-laminated retinal organoids at day (D) 105 of differentiation. Scale bar = 100 µm. **B-C:** Representative images of NR2E3 (**B**) or rhodopsin (**C**) expression in green in rosette-like structures of late-stage retinal organoids at D300 of differentiation. Nuclei are stained with Hoechst in blue. Scale bars= 50 µm.

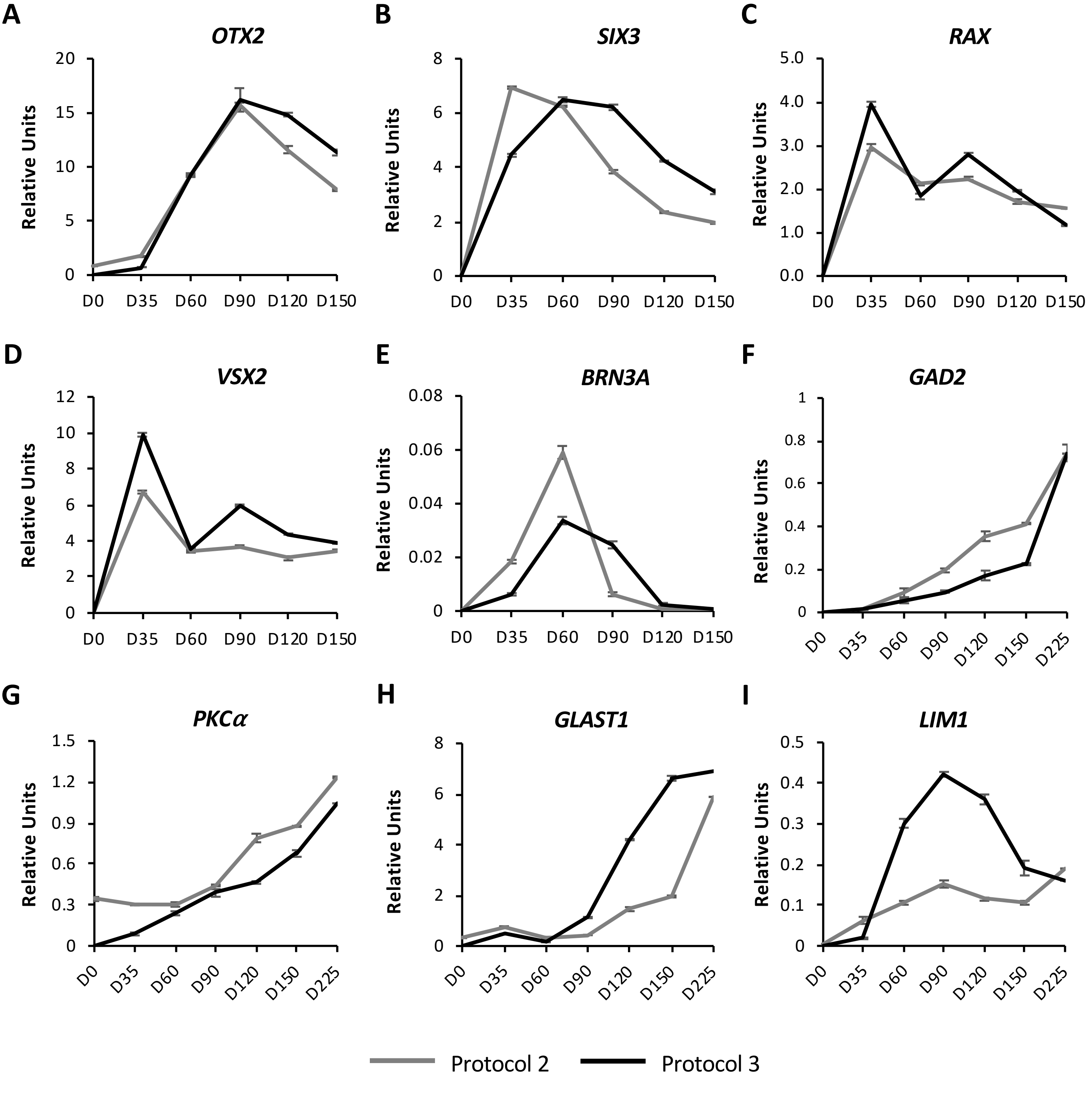


**Figure S2: Temporal gene expression of eye-field specification and non-photoreceptor markers in Protocol 2 and Protocol 3 organoids.** Pools of 25-30 retinal organoids differentiated with Protocol 2 or Protocol 3 were collected at different time points (D35, D60, D90, D120, D150 and/or D225) and relative gene expression was measured by qPCR for the eye-field specification markers: *OTX2* (**A**), *SIX3* (**B**), *RAX* (**C**) and *VSX2* (**D**). Relative gene expression was also measured for the retinal ganglion cell marker *BRN3A* (**E**), the amacrine cell marker *GAD2* (**F**), the bipolar cell marker *PKCα* (**G**), the Müller cell marker *GLAST1* (**H**) and the horizontal cell marker *LIM1* (**I**). Grey lines represent Protocol 2 and black lines Protocol 3. Data is normalised to the housekeeping gene *GAPDH* and shown as mean ± SEM, n=3.

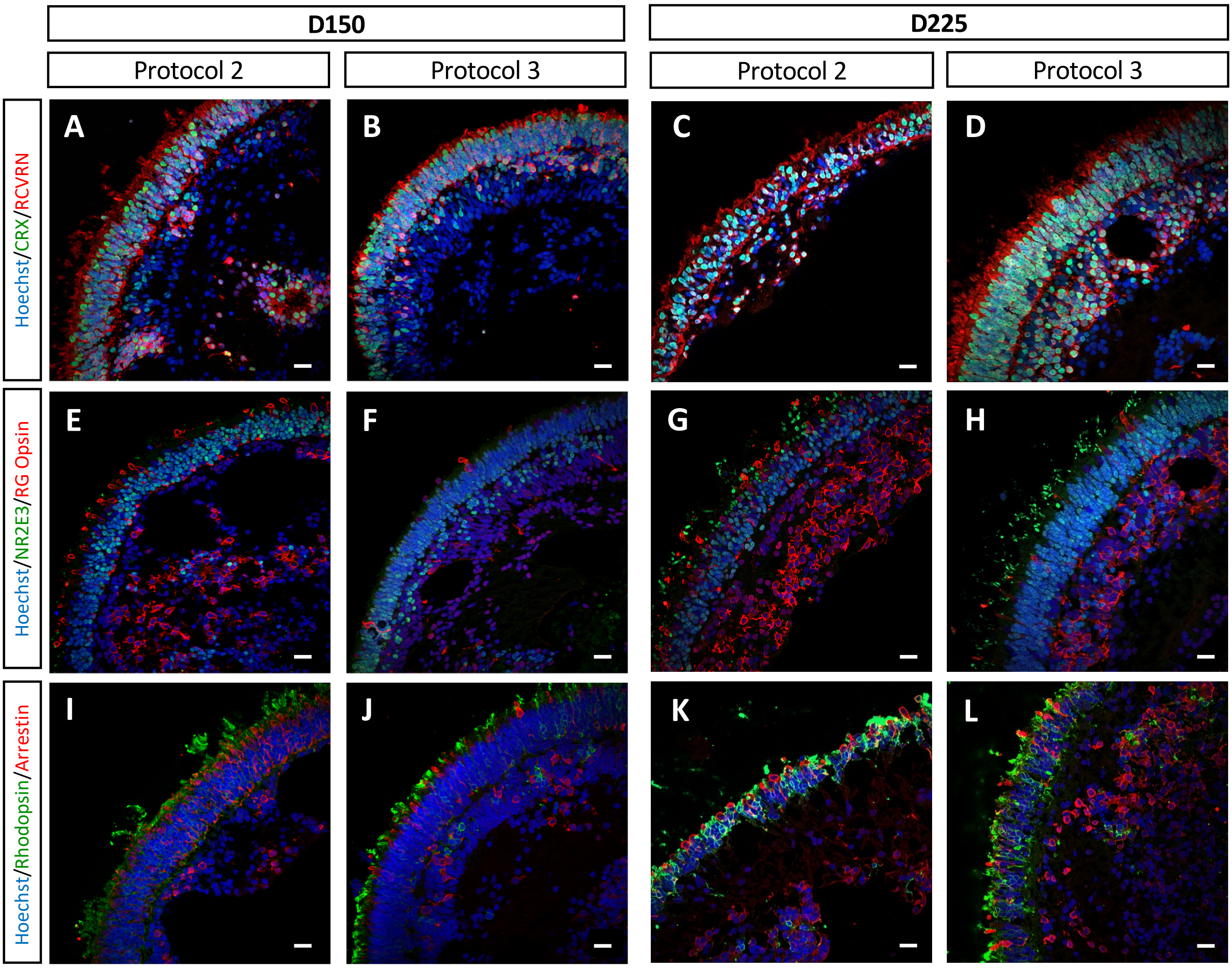


**Figure S3: Immunofluorescence studies of Protocol 2 and Protocol 3 organoids.** The same images shown in Figure 3 with the additional channel showing the Hoechst-stained ONL (in blue). Retinal organoids were analysed at D150 (**A**, **B**, **E**, **F**, **I**, **J**) and D225 (**C**, **D**, **G**, **H**, **K**, **L**) of differentiation. CRX in green and RCVRN in red in organoids generated with Protocol 2 (**A**, **C**) and Protocol 3 (**B**, **D**). NR2E3 in green and RG opsin in red in organoids generated with Protocol 2 (**E**, **G**) and Protocol 3 (**F**, **H**). Rhodopsin in green and arrestin in red in organoids generated with Protocol 2 (**I**, **K**) and Protocol 3 (**J**, **L**). Scale bars = 20 µm.

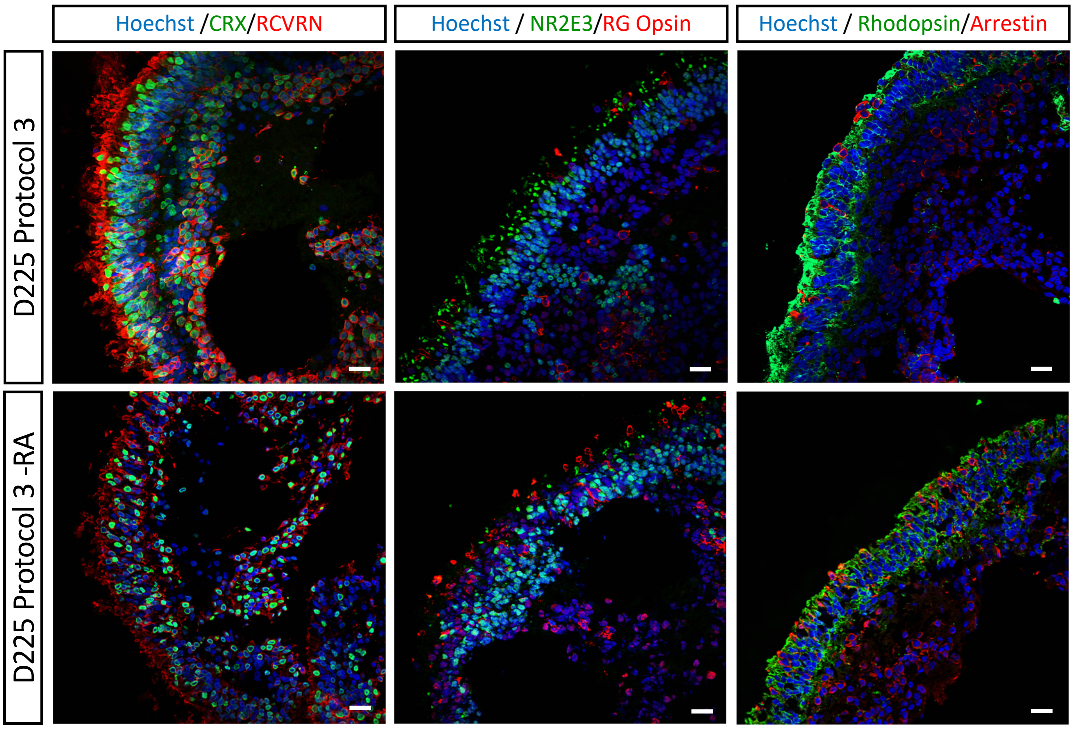


**Figure S4: Immunofluorescence studies of Protocol 3 organoids with or without RA.** The same images shown in Figure 4 with the additional channel showing the Hoechst-stained ONL (in blue). Left-hand panels, CRX in green and RCVRN in red; middle panels, NR2E3 in green and RG opsin in red; right-hand panels, rhodopsin in green and arrestin in red. Scale bars = 20 µm.

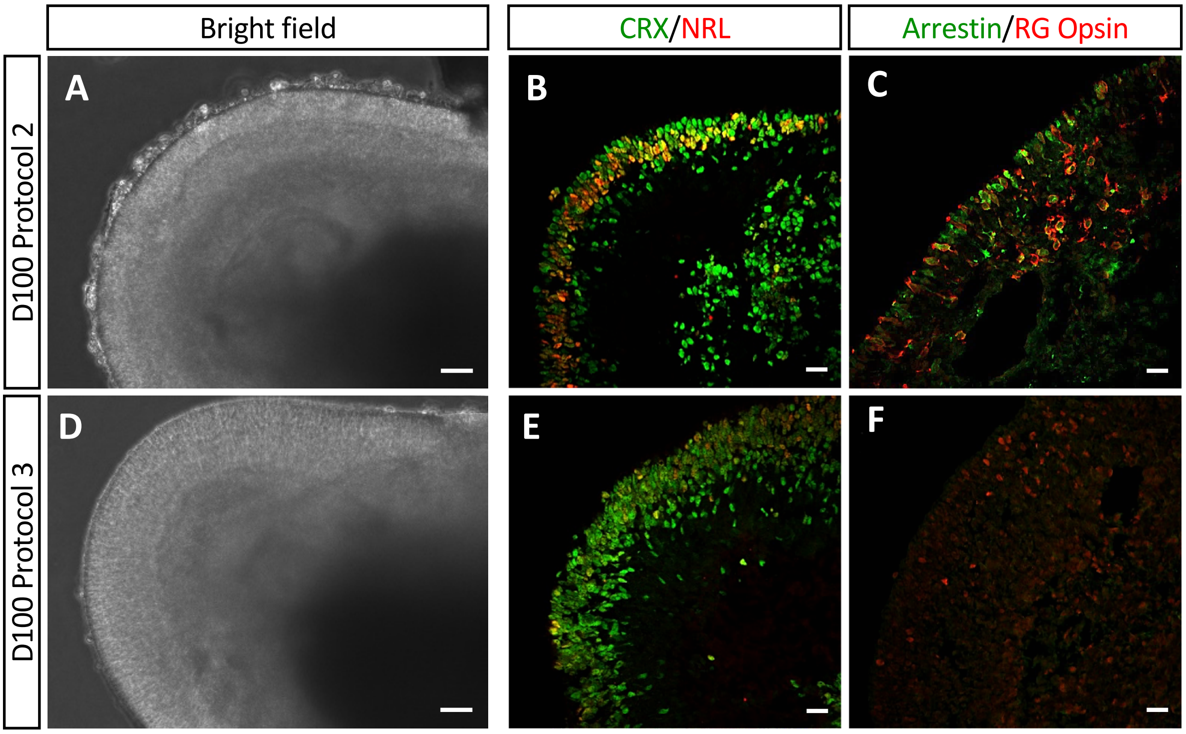


**Figure S5: Cones in early-stage Protocol 2 and Protocol 3 organoids.** Representative bright-field images of retinal organoids at D100 differentiated with Protocol 2 (**A**) or Protocol 3 (**D**). Scale bars = 50 µm. Representative IF images of CRX in green and NRL in red in Protocol 2 (**B**) and Protocol 3 (**E**) organoids. Representative IF images of arrestin in green and RG opsin in red in Protocol 2 (**C**) and Protocol 3 (**F**) organoids. Scale bars = 20 µm.
